# Gastrointestinal complications after fast-track total hip and knee replacement: an observational study in a consecutive 36,932 patient cohort

**DOI:** 10.1007/s00402-023-04887-x

**Published:** 2023-04-26

**Authors:** Louise O. H. Daugberg, Henrik Kehlet, Pelle B. Petersen, Thomas Jakobsen, Christoffer C. Jørgensen, The Lundbeck Foundation Centre for Fast-track Hip and Knee Replacement collaborative Group, Frank Madsen, Torben Bæk Hansen, Kirill Gromov, Lars Tambour Hansen, Claus Varnum, Mikkel Rathsach Andersen, Niels Harry Krarup, Søren Overgaard

**Affiliations:** 1 Department of Orthopedic Surgery, Aalborg University Hospital, Hobrovej 18-22, 9000 Aalborg, Denmark; 2 Section of Surgical Pathophysiology, 7621, Copenhagen University Hospital, Rigshospitalet, Blegdamsvej 9, 2100 Copenhagen, Denmark; 3 Lundbeck Foundation Centre for Fast-track Hip and Knee Arthroplasty, Copenhagen, Denmark

**Keywords:** THA, TKA, Fast-track, GI-complications, POI, GI-bleed, C. difficile

## Abstract

**Introduction:**

Gastrointestinal complications after total hip (THA) and knee arthroplasty (TKA) have been reported to be between 0.3 and 2.6% with bleeding and C. difficile infection in 0–1%, and 0.1–1.7%, respectively. The use of enhanced recovery or “fast-track” protocols have focused on optimizing all aspects of perioperative care resulting in reduced length of hospital stay (LOS) and potentially also gastrointestinal complications. This study is a detailed analysis on the occurrence of postoperative gastrointestinal complications resulting in increased hospital stay or readmissions in a large consecutive cohort of fast-track THA and TKA with complete 90 days follow-up.

**Materials and methods:**

This is an observational study on a consecutive cohort of primary unilateral THAs and TKAs performed between January 2010 and August 2017 in nine Danish high-volume fast-track centers. Discharge summaries and relevant patient records were reviewed in patients with readmissions within 90 days or LOS > 4 days caused by gastrointestinal complications.

**Results:**

The cohort included 36,932 patients with 58.3% females and 54.1% THAs. Mean age and BMI were 68 years and 28. Median postoperative LOS was 2 days. Only n: 276 (0.75 %) had a LOS > 4 days or a readmission within 90 days due to a gastrointestinal complication (CI 0.67%–0.84%). Of these, only 34 (0.09%) were graded as severe ileus or gastrointestinal bleeding.

**Conclusions:**

The risk of GI-complications within the first 90 postoperative days after fast-track THA and TKA was low (0.75%).

## Introduction

Gastrointestinal complications, including postoperative ileus (POI), gastrointestinal bleeding (GIB) and infection with Clostridium difficile are rare but potentially serious adverse events after total hip (THA) and knee arthroplasty (TKA). In a recent review on gastrointestinal complications after THA and TKA the overall incidence of complications was 0.3–2.6%, with GIB and C. difficile infection between 0–1% and 0.1–1.7%, respectively [1]. However, the included studies were often large database studies using administrative data [2–5] with no details on perioperative care and with the majority of data being collected before 2010 [1] thereby limiting interpretation in relation to contemporary fast-track protocols with short length of stay and fewer complications [6]. Thus, the risk of POI may be related to opioid-induced constipation [7] while GIB may be related to type and duration of postoperative thromboprophylaxis [8] as well as non-steroidal anti-inflammatory drugs (NSAIDs) [9] and C. difficile infections to type of antibiotics and extended stay in hospital [10]. In this context, the use of enhanced recovery or fast-track protocols have focused on optimizing all aspects of perioperative care, including use of opioid-sparing multimodal analgesia with NSAIDs/Cox-2 inhibitors, early mobilization, and in-hospital only thromboprophylaxis, resulting in reduced postoperative morbidity and consequently shorter length of hospital stay (LOS) [6]. It could be speculated that the use of enhanced recovery protocols may also reduce the incidence of gastrointestinal complications after THA and TKA, but detailed large-scale data on gastrointestinal complications after THA and TKA with a fully implemented enhanced recovery protocol is limited.

The aim of the present study was to analyze the occurrence of postoperative gastrointestinal complications resulting in either a LOS > 4 days or readmission within 90-days after fast-track THA and TKA in a large consecutive cohort with complete 90 days follow-up.

## Methods

### Study design

This is an observational study based on a consecutive cohort of fast-track THAs and TKAs performed between January 2010 and August 2017 in nine centers contributing to the Centre for Fast-track Total Hip and Knee Replacement database [11]. The findings of this study are presented in accordance with the STROBE statement [12].

### Data source

The Centre for Fast-track Hip and Knee replacement has since 2009 functioned as a multi-center collaboration of Danish high-volume departments with well-established fast-track protocols, including preference for spinal anesthesia, multi-modal opioid-sparing analgesia, early mobilization, only in-hospital thromboprophylaxis if LOS ≤ 5 days, and discharge to own home based on functional discharge criteria. All patients completed a questionnaire on demographic information and comorbidities prior to surgery, with nurse assistance if necessary.

Information on use of anticoagulants, psychopharmacological treatment, and antidiabetics was acquired through the National Danish National Database on Reimbursed Prescriptions (DNDRP). The DNDRP collects data on all prescriptions with reimbursement dispensed at Danish pharmacies and has shown a high degree (> 85%) of completeness for most drugs [13].

Data on LOS, 90-days readmissions and mortality was retrieved from the Danish National Patient Registry (DNPR). The DNPR collects information on all somatic admissions in both the public and private sector, ensuring follow-up of > 99% [14].

The medical discharge summary was reviewed in cases of LOS > 4 days or 90-day readmission to identify the reason for admission. In case of insufficient information and in all cases of mortality, the entire medical record was reviewed.

We secondarily reviewed the complete medical records of all patients with initially recorded gastrointestinal complications for information on onset and severity. In case of GI-complications during primary admission without well-defined time of onset, but subsequent endoscopy or transfer to surgical ward we used onset as day of procedure/transfer. In case of readmission, we defined onset as day of readmission.

In 20 (16.4 % of GI-complications during primary admission) cases of GI-complications during primary admission it was not possible to retrieve information on onset of symptoms, day of performed procedure or transfer to a surgical ward.

### Study cohort

The cohort included consecutive elective unilateral primary THAs and TKAs performed between January 2010 and August 2017 [11] with a completed preoperative questionnaire. We excluded patients < 18 years, those with prior major lower extremity surgery within 90 days of the THA or TKA procedure, simultaneous bilateral procedures, procedures due to fractures, cancer, or severe congenital disorder.

### Assessments

GIB was defined according to the European Perioperative Clinical Outcome definitions, where gastrointestinal bleeding is an “unambiguous clinical or endoscopic evidence of blood in the gastrointestinal tract,” and severity is graded into mild (temporary harm, usually no specific treatment), moderate (no permanent harm but usually requires treatment), and severe (significantly prolonged LOS and/or permanent functional limitation or death, always requires treatment) [15].

POI was graded according to Venara et al classification proposed in 2017. The grades are A (no consequence of POI except an increase in LOS), B (need for treatments such as laxatives, etc. or diagnostic examination), C (need for nasogastric tube or readmission), D1 (systemic complication, e.g., ionic imbalance), D2 (ICU or further surgery) and E (death) [16].

### Statistics

The frequency and percentage of each included gastrointestinal complication was calculated in SPSS Statistics 28.0 by IBM. 95% Confidence Intervals (CI) for proportions were calculated with the use of Vassarstats.net.

### Ethics and permits

This study has been approved by the National Board of Health (3-3013-56/1/), the Capital Region of Denmark (R-20073405) and the center for Regional Development (RH-2007-30-0623). The National Committee on Health Research Ethics waivered the need for permission as this was an observational study.

## Results

The mean age was 68 years (SD: 10.6), mean BMI was 28 (SD: 5.2), 54.1% had THA and 58.3% were females (Table 1). The median postoperative LOS was 2 days and time from surgery to onset of GI-complication was overall a median of 6 (IQR: 3–23) days, but 2 (IQR: 1–3) days for complications during primary admission and 19 (IQR: 7–36.8) days at readmission.

**Table 1:** Patient characteristics

Category	Subcategory	*N* (%)
THA	Yes	19,976 (54.1)
Female	Yes	21,523 (58.3)
Age	< 50	2019 (5.5)
	50–60	5992 (16.2)
	61–65	5183 (14.0)
	66–70	7418 (20.1)
	71–75	7193 (19.5)
	76–80	5342 (14.5)
	81–85	2733 (7.4)
	> 85	1052 (2.8)
BMI	< 18.5	284 (0.8)
	18.5–24.99	10,119 (27.4)
	25.00–29.99	14,520 (39.3)
	30.00–34.99	7999 (21.7)
	35.00–39.99	2746 (7.4)
	> 39.99	1017 (2.8)
Living situation	With others	24,264 (65.7)
	Alone	12,251 (33.2)
	Institution	243 (0.7)
Smokers	Yes	5080 (13.8)
Abstains from alcohol	Yes	2760 (7.5)
Walking aid	Yes	8468 (22.9)
Comorbidities	Cardiac	5070 (13.7)
	Pulmonary	3234 (8.8)
Anemia (Hgb < 13 g/dL)*	Yes	8921 (24.2)
Diabetes	Insulin dependent diabetes	837 (2.3)
	Oral antidiabetics	2615 (7.1)
	Diet controlled	566 (1.5)
Potent anticoagulants	Yes	2538 (6.9)

*Anemia was defined as Hgb < 13 g/dL due to newer literature suggesting updating the WHO criteria for anemia to < 13 g/dL in both sexes would be beneficial as pertaining to surgery [28, 29]

Out of the 36,932 included fast-track THA and TKA procedures a total of 276 (0.75%) experienced GI-complications. Of these 123 (0.33%) resulted in a LOS > 4 days and 153 (0.42%) in readmission within 90 days. The primary incidence of GIB, POI and C. difficile was 37 (0.1%), 19 (0.05%) and 2 (0.01%), while the incidences of these at readmission were 46 (0.12%), 9 (0.02%) and 11 (0.03%).

Of the 83 (0.22%) patients who experienced GIB, 17 (0.05%), 49 (0.13%) and 17 (0.05%) experienced mild, moderate, and severe bleeding, respectively. Out of the 83 patients that experienced a GIB, only 8 took potent anticoagulants. The respective mean LOS at primary admission and readmission was 5.3 days and 6.4 days.

Of the 28 (0.07%) patients that experienced POI 2 (0.01%), 5 (0.01%), 4 (0.01%), 13 (0.04%) and 4 (0.01%) were graded as A, B, C, D2 and E. The respective mean LOS at primary admission and readmission was 5.0 days and 5.2 days.

For information on the incidences of the other included gastrointestinal complications see Table 2.

**Table 2:** LOS, readmissions, and complications

Category	Subcategory	*N* (%)
LOS > 4		2391 (6.5)
Readmission within 90 days		2824 (7.6)
GI-complications during initial hospitalization*		123 (0.33)
	GIB	37 (0.1)
	Gastritis	23 (0.06)
	Constipation	21 (0.06)
	POI	19 (0.05)
	Diarrhea	9 (0.02)
	Pancreatitis	4 (0.01)
	Diverticulitis	3 (0.01)
	Liver failure	3 (0.01)
	C. difficile*	2 (0.01)
	Abdominalia	2 (0.01)
GI-complications triggering readmission		153 (0.41)
	GIB	46 (0.12)
	Constipation	31 (0.08)
	Diarrhea	28 (0.08)
	Gastritis	19 (0.05)
	C. difficile*	11 (0.03)
	POI	9 (0.02)
	Abdominalia	9 (0.02)
	Diverticulitis	1 (0.0)
Ileus grade**	A	2 (0.01)
	B	5 (0.01)
	C	4 (0.01)
	D2	13 (0.04)
	E	4 (0.01)
GIB grade***	Mild	17 (0.05)
	Moderate	49 (0.13)
	Severe	17 (0.05)

*Confirmed C. difficile infections

**According to Venara et al. [16]

***According to EPCO, 2015[15]

## Discussion

This study on GI-complications after fast-track THA and TKA found lower rates of POI, GIB and C. Difficile infections than previously reported. A comparable study from the US [17], including > 1.5 million THA and TKA patients and reporting a declining incidence of GI-complications between 2006 and 2016 found an incidence of acute GI-complications of 1.03% and 0.79% in THA and TKA, respectively compared with a 90-day incidence of 0.75% in total, whereof the incidences of POI, GIB, and C. difficile were 0.07%, 0.22% and 0.04%.

It has previously been theorized that the implementation of enhanced recovery pathways may reduce the number of GI-complications [17], as several elements such as multimodal analgesia, early mobilization, only in-hospital thromboprophylaxis and reduced need of hospitalization may reduce the risks of GI-complications such as GIB and constipation [17–19] as demonstrated in our study.

Thromboprophylaxis after THA and TKA surgery is a complicated task due to the opposite risks of VTE and GIB, which was the most common GI-complication in our cohort. The risk of GIB after THA and TKA remained increased for 6 weeks after surgery for osteoarthritic patients in a study by Lalmohamad et al. [3]. This increased risk may be related to the use of thromboprophylaxis, although most patients received subcutaneous low molecular weight heparin, which is generally not associated with a higher risk of GIB [3]. In addition, NSAIDS, which are often used in a multi-modal opioid-sparing protocol, have not been found to increase the risk of GIBs after THA or TKA if appropriate precautions are taken as it pertains to patients with a higher risk of GIB [19]. The individual pain treatment plans and use of proton-pump inhibitors were not reviewed in this study.

The low incidence of GIB in this study may also be related to a short duration of thromboprophylaxis in most Danish THA and TKA patients who receive only in-hospital thromboprophylaxis when LOS is ≤ 5 days. [20]. Although such a thromboprophylaxis regimen contest most international guidelines, which recommend thromboprophylaxis for 10–35 days, the safety of in-hospital only thromboprophylaxis in fast-track THA and TKA has previously been documented [20].

THA and TKA along with an elderly population, immobility, and side effects of medication such as opioids may lead to constipation and ileus [21]. A very low number of the included patients in our study experienced clinically relevant constipation—52 patients (0.14%) compared to up to 65% in a RCT by Ross-Adijie et al. [22]. This finding makes sense in a fast-track enhanced recovery setting, since the protocol ensures early mobilization, minimal use of opioids potentially reducing the risk of constipation.

POI is a complication that occurs with an incidence of 0–2.1% [1] or 0.3–4% [23] in THA and TKA. In this study, only 28 patients (0.08 %) experienced ileus. The main risk factors of POI have been found to be older age, male gender, hip arthroplasty, chronic kidney disease, prior history of myocardial infarction and/or previous abdominal surgery [23, 24]. Despite that these risk factors are difficult to modify, fast-track surgery does not seem to increase the risk of POI. The results of this study may even suggest that it decreases the risk of POI.

The incidence of C. Difficile has been found to between 0 and 1.7% after THA and TKA surgery [1]. Antibiotics are used to prevent deep joint infection after surgery [25], and meta-analyses have found broad spectrum antibiotics, especially cephalosporins and clindamycin, to increase the risk of C. difficile infection 2-3-fold [26]. The increased risk of contracting C. difficile remains for up to 90 days. However, in a study by Al-Tawil et al. the use of 3 peri- and postoperative doses of cefuroxime did not appear to be associated with an increase in C. difficile rates [26]. In this study, it was not possible to specify which antibiotics were used during the 7-year period in the 9 included high-volume centers since each region followed individual guidelines. Out of the included patients in our study, 13 had negative C. difficile toxin tests and six patients only had nausea, corresponding to a maximum of 29 (0.08%) with potential C. difficile infection within the 90 postoperative days, matching the incidences of most studies on this subject [1, 27]

The main limitation of this study is that many minor to moderate GI-complications such as nausea might not have been mentioned in patients’ medical records and thereby not included. Pertaining thereto, any GI complication that did not result in a LOS > 4 days or a readmission within 90 days was not included. Yet, it can be argued that all clinically relevant GI-complications have been included since all patients with an acknowledged GI-complication have had their records and discharge resumes read and evaluated by the author group. Another limitation is the observational study design which may provide associations, but not causation, that the use of an enhanced recovery protocol may reduce occurrence of GI-complications. In this context, the lack of a control group not having fast-track surgery also hinders direct comparisons. The strengths of this study include that the cohort accounts for about 50% of the Danish THA and TKA production [11]. Due to the Danish tax-based healthcare system discharged patients would not have an economic reason to remain at home if faced with GI-complications, wherefore the results may provide a realistic incidence estimate.

## Conclusion

We found an incidence of GI-complications after fast-track THA and TKA to be 0.75% within the first 90 postoperative days. The incidences of POI, GIB, and C. difficile were 0.07%, 0.22% and 0.04%, respectively. These low occurrences of gastrointestinal complications could be related to the use of established enhanced recovery protocols with early mobilization, opioid sparing analgesia and only in-hospital thromboprophylaxis if LOS ≤5 days.

## Data Availability

Anonymous data are available from the authors upon request.
